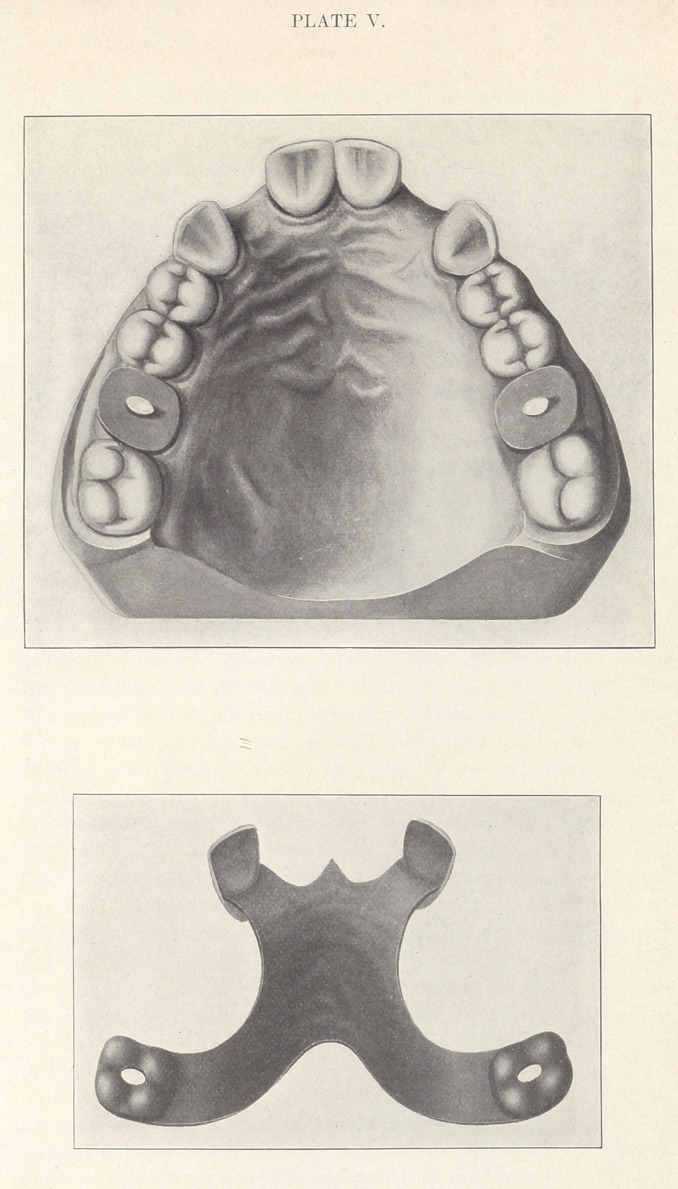# The Burnished-Cap-Crown

**Published:** 1902-08

**Authors:** P. B. M’Cullough

**Affiliations:** Philadelphia


### International Dental Journal.

Vol. XXIII. August, 1902. No. 8.

### Original Communications.[1]

### "THE BURNISHED-CAP-CROWN."[2]

BY DR. P. B. M'CULLOUGH, PHILADELPHIA.

Mr. President, I have the honor to offer for the consideration of this Academy a description of the method for making "The Burnished-Cap-Crown."

Plate I. For the best application of this system a set of instruments are required consisting of eighteen impression-tubes like Figs. 1 to 5, each provided with an ejector; a handle having two curved bite plates attached, and four sockets at different angles for the reception of the nozzle end of the tubes. (Fig. 6.)

A swaging device in two parts, consisting of a mandrel (Fig. 7) and a matrix plate (Fig. 8), the latter serving also as a model support, Figs, 6, 7, and 8 being sectional views.

Preparatory to crowning any of the upper six anterior teeth, the root is ground slightly below the gum margin following the festoon; this will give a curve mesio-distally and a straight line labio-palatally; if any enamel remains it is chipped off and the root bevelled with a cone bur.

[1] The editor and publishers are not responsible for the views of authors of papers published in this department, nor for any claim to novelty, or otherwise, that may be made by them. No papers will be received for this department that have appeared in any other journal published in the country.

[2] Read before the Academy of Stomatology, Philadelphia, December 27, 1901.

The impression-tube best suited to the case is selected, placed in the socket in the end of the handle provided for the upper anterior teeth, filled with zinc phosphate, and pressed to place upon the root, care being taken that the tube be held fixed until the cement becomes hard.

By holding the tube together with a corner of a piece of paper the size of a prescription blank between the thumb and fingers of one hand, and turning until a cornucopia is formed which when bound with waxed thread and trimmed with scissors until walls have a depth of a quarter of an inch around impression, constitutes the matrix. In the pocket thus formed is dropped from tweezers a mixture of prepared chalk in dilute sandarach, in the proportion of one-third precipitate when settled and immediately shaken off, so as to form a very faint and even coating over the impression. when cement mixed thick is packed in the matrix. After unwinding the string and paper the model and impression are pulled apart, the two being readily separated.

PLATE II. After trimming the model (Fig. 1) with a coarse corundum stone dry without injury to that part, reproducing face of root and bevel, it is waxed to the flat surface of the model support. A piece of 36-gauge platina plate is then held firmly upon the face of the model with the thumb of one hand and the edges turned down on the bevel with the blade of a No. 8 ball burnisher in the other, until an approximately fitting cap is formed. This is then laid aside, and a gold band of 22-carat, 28-gauge, one-eighth of an inch wide, cut with angular ends, is measured around the model, the ends soldered and shaped upon the model, the cap annealed, returned to place, the band pressed over cap, drawing it tight to bevel, when both are removed adherent and soldered together from the side to which the porcelain is to be adjusted, using sufficient solder to cover the cap. (Fig. 2.)

The extended platina edges are then cut away, the part fitting under the gum bevelled, the surface to which the porcelain is to be fitted filed flat at an angle, so that the band will be widest on its palatal and narrowest on its labial face. (Fig. 3.)

From seven-eighths of an inch of No. 18 iridio-platina round wire the dowel is made by filing flat at an angle one side extending from the end one-fourth of an inch; this is cut off and clamped to the five-eighths-inch piece, flat side in, soldered, and filed to the shape of a flattened obelisk.

PLATE I.

Fig. 1.

Fig. 2.

Fig. 3.

Fig. 4.

Fig. 5.

Fig. 6.

Fig. 7.

Fig. 8.

PLATE II.

FIG. 1.

FIG. 2.

FIG. 3.

FIG. 4.

FIG. 5.

A hole punched in the centre of the cap is filed to shape of dowel large enough to admit the latter for half its length, so that the edges of the hole may not be turned back when the dowel is forced to place.

Thus the two sections are ready to be placed in position upon the root, when, by virtue of the particular shape of the dowel and the cap stiffened with solder, they are withdrawn adherent and soldered without investing and returned immediately to place for the impression in plaster, with, preferably, No. 20 tray.

After the facing has been ground flat to fit the cap, and before the cut pins are split to secure the thick platina backing, No. 20 or 60 pure gold should be caught by an edge between the backing and the tooth and smoothed over the ground end fitting the cap, to protect the porcelain from borax and reflect a better shade at the joint.

From this point the crown is finished after the accepted methods. (Fig. 4.) Usually, as a guide for fitting facing, the correct length of the crown is measured by touching a straight line connecting the cutting edges of the two adjacent natural teeth.

For the lower bicuspid the root is ground flat rather than following the festoon of the gum,—although this is optional,—and bevelled. The impression is taken with the tube in that socket of the handle at a right angle to its length.

When placed upon the root the patient is directed to close on bite plate, with sufficient pressure to assist the operator in retaining the tube fixed until the cement becomes hard. Thereupon the procedure is the same in detail as described for the incisor up to the point where the cap and dowel soldered have been returned to the root. A mix of plaster is then piled upon the cap and neighboring teeth and the patient directed to close, holding the jaws fixed until the plaster sets.

Should the plaster break upon removal it can be held together until freely coated with shellac and sandarach and both sides filled with plaster. Then by cutting away the impression-material until the teeth are exposed, the model may be pulled asunder. Thus the return of the fractured parts insures an accurate model and bite.

The use of a saddle-back tooth makes this typically a lower bicuspid crown. (Fig. 5.)

One detail worth observing is that the backing be made to extend straight out all around, except where adjusted to the cap, so that by drawing the solder over a greater area a more perfect contour is attained in finishing and a fault in the shape of these teeth partially overcome.

Plate III. For the upper bicuspid the root is ground flat and bevelled, handling the tube as described for the incisor or lower bicuspid and following in detail the work as stated for these two crowns up to the burnished cap. Then a piece of 22-carat gold, 28-gauge, three-sixteenths of an inch wide, is cut curved, with ends forming an acute angle with the convex edge (Fig. 1), so that when measured around the model and the ends soldered the ferrule will have a greater diameter at one end than at the other and set flat upon either edge.

After fitting the smaller end to the model the cap is placed upon the latter and the ferrule or band, as fitted, pressed to place, the sections removed adherent, and soldered. The section is then returned to the model, the walls pressed out mesially and distally for approximal contact, and in imitation of the outline of a natural bicuspid, as seen looking towards its occlusal surface.

With dowels made as described for the other crowns, and holes made in the cap, if a bicuspid with two canals, it is placed upon the root, observing that the edge of the band touches the tooth front and back of the space. One dowel is then pressed to place, removed with the cap, soldered, and the operation repeated for the other.

If the edge of the band on the palatal surface extends out of line of the adjacent teeth it is cut out, forming a curve to the depth required, beginning mesially at the point of juncture of the palatal with the mesial wall, and terminating at an opposite point distally. (Fig. 2.) If out of line on the buccal face, it is split, the edges lapped and pressed into alignment while upon the root. The band is then filled to excess with zinc phosphate and the patient directed to close the mouth, while the operator marks a straight line on the cement connecting the buccal cusps of the two adjacent natural teeth, thus marking the lateral occlusion, which, together with the bite, constitutes the articulation.

The excess of cement is then ground off with a coarse corundum stone, dry, and the cusps carved after a natural bicuspid, exposing the edge of the band all around (Fig. 3), when it is embedded in hard wax in the mandril section of the swaging device, leaving the cement and edge of the band exposed.

Over the modelling clay or mouldine filling the hole in the matrix plate is laid a piece of gold 22-carat, 28- or 30-gauge, and

### PLATE III.

Fig. 1.

Fig. 2.

Fig. 3.

Fig. 4.

Fig. 5.

Fig. 6.

Fig. 7.

PLATE IV.

Fig. 1.

Fig. 2.

Fig. 3.

Fig. 4.

the cement cusps held upon the centre and driven into the gold with horn mallet, annealing, trimming, and pressing the mouldine back in the plate as required, until the swaged section is formed to the model and to the edge of the band. (Fig. 4.)

The wax is then softened, the section embedded removed, a flame from the blow-pipe directed on the cement, which is immediately dropped in hydrochloric acid and repeated, if necessary, until the cement is destroyed.

The cusp section, pickled and stiffened with 20-carat solder, is wired and soldered to the band which it will fit as swaged by virtue of the curve in the cusp section corresponding with the curve in the band.

Thus a hollow crown is formed, the buccal face of which is sawed out and the remaining shell filled with 16-carat solder over a Bunsen flame. (Fig. 5.)

Flat surfaces are then filed to receive the facing, the ends of which are ground flat at the required angles to fit the space, after the fashion of an inlay, and backed with pure gold with the ends extending beyond the sides of the facing, and held by splitting the previously cut pins. The facing is then wired in place, protected with four-ply asbestos cloth, wired, wet and packed with the fingers. (Fig. 6.) Upon one end of the extended backing is placed 14-carat solder and held over a Bunsen flame with facing downward, at an angle so as to favor the gravitation of the solder, when melted, through the small space between the backing and the body of the crown to the other side.

The only facing made fit for this crown is one having thick, flat, angular ends. By a fault of them all being too long to imitate a natural crown, and their little shape destroyed by grinding, it is often necessary to have the porcelain extend out beyond the gold, forming the buccal cusp, so that when finishing the natural curve may be formed from the point of the cusp to the edge of the cap. (Fig. 7.)

The lower molar is ground, curved, following the line of the gum, and bevelled, the tube fixed in one of the two sockets in the handle provided for the molars, and the operator assisted by pressure of the upper teeth on the bite plate while taking the impression.

The detail of construction is the same as described for the upper bicuspid, up to the point where the cap and band are soldered together. With this section in place on model, the free edge of the band constituting the walls of the crown is pressed out all around after the outline of the natural tooth as seen looking towards its occlusal surface.

It may be observed that the walls of a natural lower molar crown diverge from the line of attachment of the enamel to the cementum for two-thirds its total depth on the mesial, lingual, and distal surfaces, and one-third on its buccal face; thus the converging lines terminating in the cusps form two-thirds the depth of the buccal face and one-third of each of the other three surfaces.

The gold section, constructed to the point described, has a band of equal width all around, representing two-thirds the depth of the finished crown. In order that all converging lines may be formed in the cusp section, half the depth of the band is cut out on its buccal face, forming a curve extending from the point of juncture of the mesial with the buccal wall and termiaating at an opposite point distally. (Plate IV., Fig. 1.) In this "curve of contour" lies the secret of the perfectly contoured crown. This section is then placed upon the root, and if the space is normal the free edge of the band should touch the approximal surfaces of the adjacent natural teeth, thus forming the interdental space; then the band is filled with an excess of cement and the patient directed to bite. The cement forced out by the occluding teeth is ground off and the piece returned to the root; the patient is then directed to bite laterally, while the cement is ground in the mouth until the proper lateral occlusion is attained.

After carving the cusps (Fig. 2) the section is waxed in the mandrel and the swaged section ( Fig. 3) formed, after the manner described for the upper bicuspid.

Before soldering the cusp section to the band the centre of the platina cap is cut out with a plate punch and filed, leaving an edge extending from the inside of the band a sixteenth of an inch all around. The object of this cap in the molar crown is to strengthen the crown at its bevelled edge and to serve as an absolute guide in "forcing" the crown to place upon the root.

This description answers for the upper molar, excepting that the band is not pressed out on its buccal face, and the curve in the band is cut on the palatal surface as described for the buccal face of the lower, and the lateral occlusion is marked in the cement by drawing a straight line connecting the buccal cusps of the two adjacent natural teeth.

The drawings for the telescoping crown (Plate V.) illustrate a practical case as finished, showing the cap and post section cemented upon the root of each of the upper first molars, and the telescoping section soldered to a gold plate, supporting two lateral incisors.

*The Telescoping Crown.*—The root for the upper molar is ground flat and bevelled, the walls of the pulp-cavity made parallel, in which, to provide the necessary anchorage, is fitted a tube approximating the shape of the cavity walls and resting on the floor of the cavity, with the other end extending beyond the edge of the root a sixteenth of an inch. The tube is then set with plaster, the latter trimmed flat and even with the ground edge of the tooth, with the tube extending out of the centre. An impression is then taken with cement, with the proper impression-tube, withdrawn with tube embedded, wrapped with paper forming a matrix, coated with separating medium, and packed with cement. When the impression-material is cut away the tube will be embedded in the cement model and extending out of the centre as out of the pulp cavity in the mouth.

With the model waxed to the model support, a hole is cut in the centre of a piece of 36-gauge platina plate large enough to admit the extending end of the tube and rest flat on the model; the tube is then filed off flush with the platina cap and the latter turned down on the bevel of the model with a burnisher. A 22-carat gold band, 28-gauge, an eighth of an inch wide, is then measured around the model, the ends soldered, shaped upon the model, and pressed to place over the platina cap, drawing it tight to bevel. The cement model, holding the tube, cap, and band, is then placed upon the block and the three sections soldered together. A piece of 28-gauge, 22-carat plate is then fitted on the cap inside of the band, soldered, and the entire surface filed flat, including the edge of the band, thus forming a solid flat gold covering over the platina cap. The cement is broken up with fire and acid, when the section is ready for the patient. If a case like the one illustrated, then, of course, the operation as described is duplicated.

With both sections placed upon the roots, an impression of all the teeth is taken in plaster, the sections placed in position in the impression, and a model made with plaster and sand. A plaster model is made from a plaster impression of the lower jaw and both models fixed in an articulator.

A method for forming the telescoping tubes, the inner constituting the finished post and the outer a part of the movable section, may be done by cutting a piece of clasp gold, 30-gauge, measuring half an inch square, and shaping on a round instrument of suitable size until two edges meet; when soldered this will give a tube of even diameter throughout. Around this a piece of the same metal one-quarter of an inch wide, long enough for the ends to meet, is moulded and the edges soldered; the two are then forced together and a round instrument, smaller than the inner tube, is placed inside and the outer tube struck with a mallet until slightly flattened; this will prevent the tubes turning one upon the other, and by shaping them together their sliding property will not be destroyed.

Each inner tube is then adjusted upon the centre of the cap on the investment model at any angle conditions might suggest, but they must be absolutely parallel; they are then waxed, held with investment material, and soldered to the cap. The tubes are then ground to fit the irregular surface of the occluding plaster tooth, the telescoping tubes placed over the first and likewise articulated. A gold band to form the walls of the finished crown is then shaped and fitted to the edge of the cap, the latter covered with thin paper to prevent the wax adhering, and the bottom of the space between the telescoping tube and the band constituting the walls of the crown is covered with hard wax, thus uniting the two. The "curve of contour" is then cut in the palatal face with a stone, the band filled with an excess of cement, and the articulator brought together. Slip off the telescoping section, carve the cement, and expose the edge of the tube in the centre of the occlusal surface, and the edge of the band all around; fix in the mandrel and swage the cusps, cutting out the centre to the edge of the tube. Then remove from the mandrel, clean off the wax, and solder the cusp section to the tube and to the band; pickle to remove the cement, and then fill the space between the tube and the walls of the crown with low-carat solder. The crowns are then set with cement in the mouth, and the swaged plate held in position, while a plaster impression is taken of one crown and a part of the plate, then invested, and the telescoping section and plate soldered together; the operation is repeated for the other side. Then an impression is taken for mounting the teeth.

In the tube, to provide anchorage, fitting the pulp-cavity, were drilled a number of holes to afford an even distribution of the cement in forcing the crown to place. Inside the tube constituting the post a hole was drilled through the cap to afford escape for excess cement, and after setting, the tube was filled with cement and covered on the exposed occlusal surface with amalgam.

The principal upon which this crown is made applies alike to every tooth in the mouth; for bicuspids and incisors the post upon which the telescoping section rides is made by soldering together two pieces of square iridio-platina wire, which may be separated at the free end for "spring," but in every case, to be typical, the post must extend through the crown to the occlusal surface. In an incisor crown the post would run next the backing of the facing.

## Figures and Tables

**PLATE I. f1:**
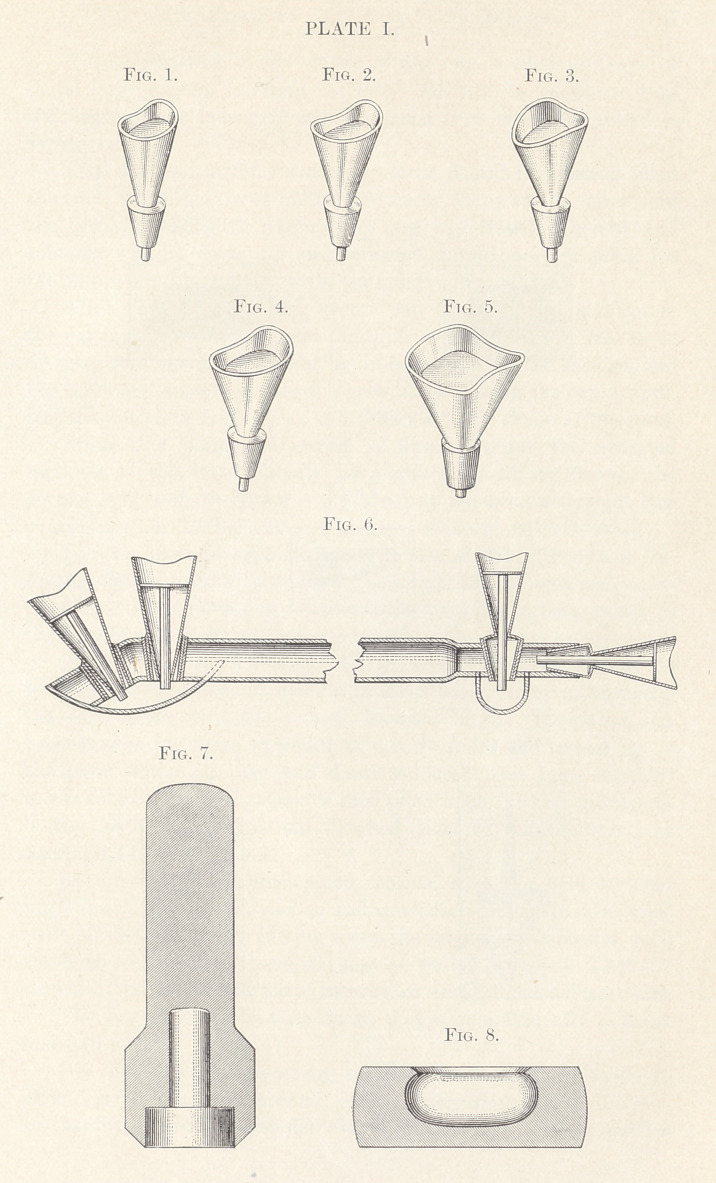


**PLATE II. f2:**
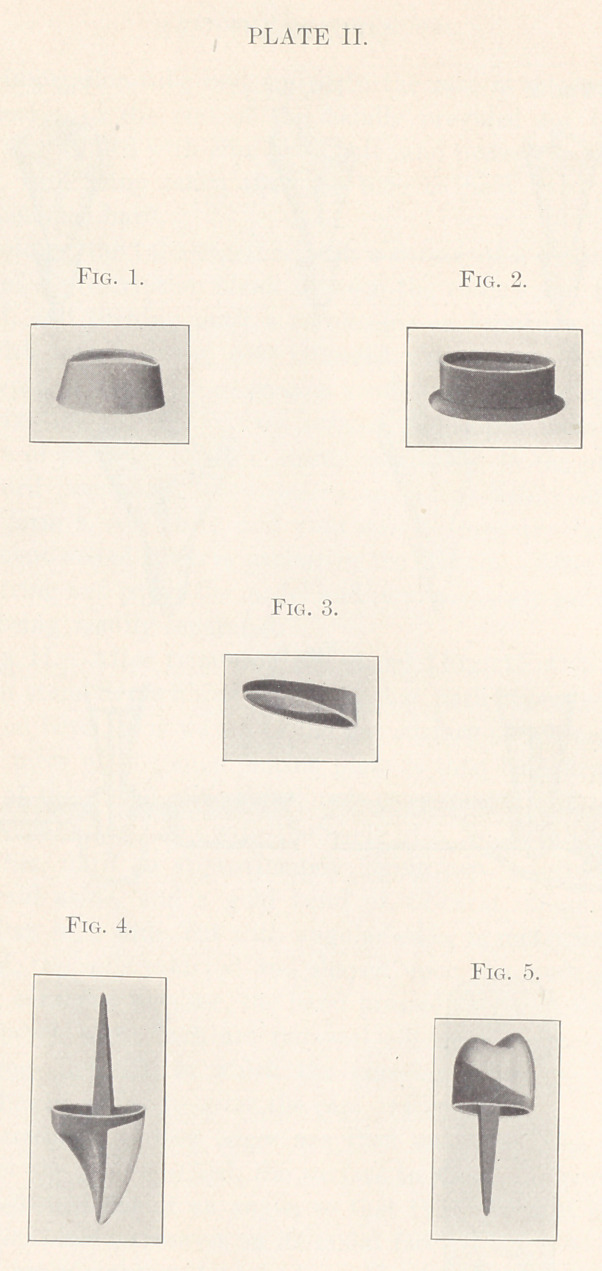


**PLATE III. f3:**
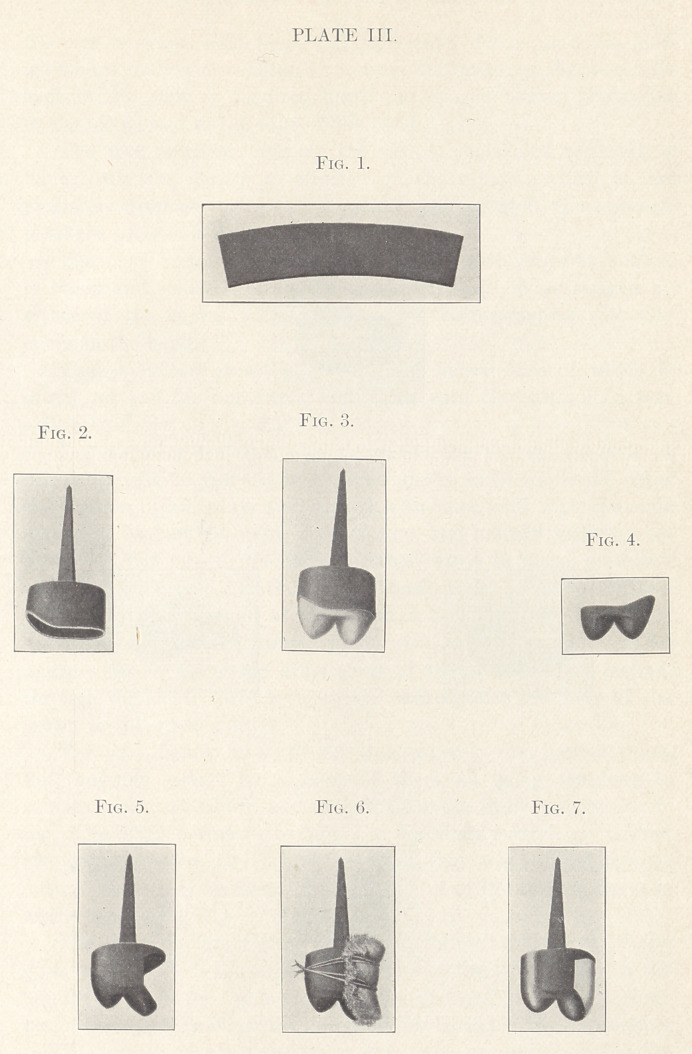


**PLATE IV. f4:**
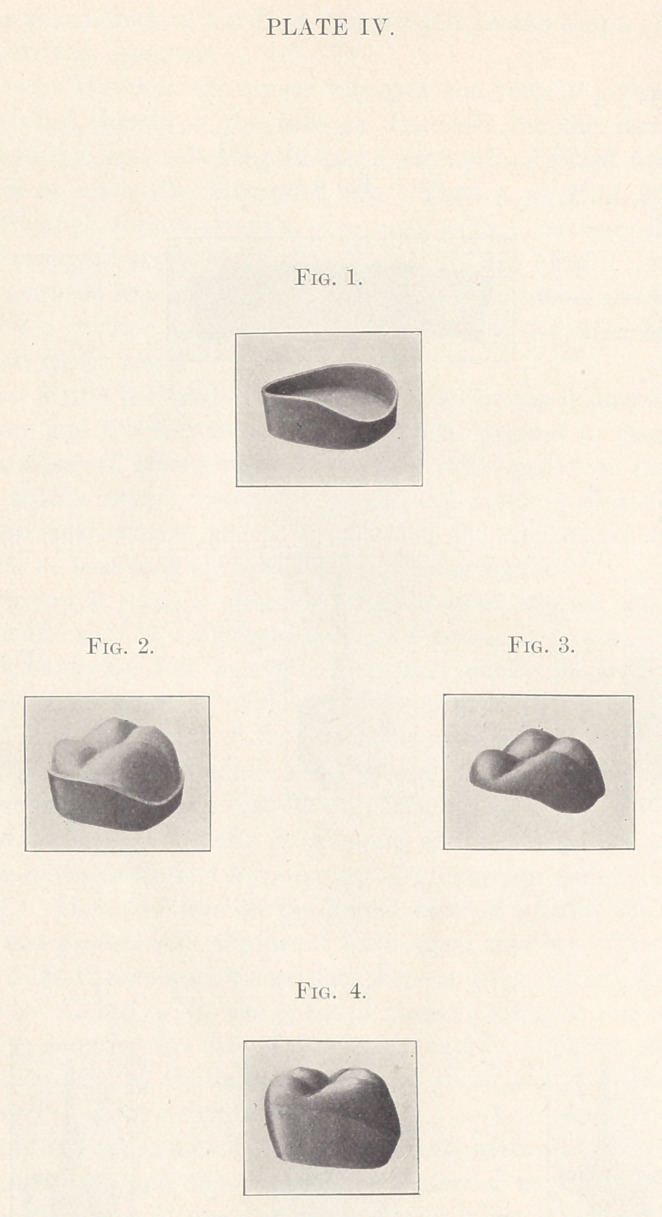


**PLATE V. f5:**